# Improved U-net-based leukocyte segmentation method

**DOI:** 10.1117/1.JBO.28.4.045002

**Published:** 2023-04-12

**Authors:** Mengjing Zhu, Wei Chen, Yi Sun, Zhaohui Li

**Affiliations:** a Xi’an University of Science and Technology, School of Communication and Information Engineering, Xi’an, China; b Xi’an Key Laboratory of Network Convergence Communication, Xi’an, China

**Keywords:** leukocyte segmentation, U-net, retinex, attention mechanism, loss function

## Abstract

**Significance:**

Leukocytes are mainly composed of neutrophils, basophils, eosinophils, monocytes, and lymphocytes. The number and proportion of different types of leukocytes correspond to different diseases, so an accurate segmentation of each type of leukocyte is important for the diagnosis of disease. However, the acquisition of blood cell images can be affected by external environmental factors, which can lead to variable light and darkness, complex backgrounds, and poorly characterized leukocytes.

**Aim:**

To address the problem of complex blood cell images collected under different environments and the lack of obvious leukocyte features, a leukocyte segmentation method based on improved U-net is proposed.

**Approach:**

First, adaptive histogram equalization-retinex correction was introduced for data enhancement to make the leukocyte features in the blood cell images clearer. Then, to address the problem of similarity between different types of leukocytes, convolutional block attention module is added to the four skip connections of U-net to focus the features from spatial and channel aspects, so that the network can quickly locate the high-value information of features in different channels and spaces. It avoids the problem of large amount of repeated computation of low-value information, prevents overfitting, and improves the training efficiency and generalization ability of the network. Finally, to solve the problem of class imbalance in blood cell images and to better segment the cytoplasm of leukocytes, a loss function combining focal loss and Dice loss is proposed.

**Results:**

We use the BCISC public dataset to verify the effectiveness of the proposed method. The segmentation of multiple leukocytes using the method of this paper can achieve 99.53% accuracy and 91.89% mIoU.

**Conclusions:**

The experimental results show that the method achieves good segmentation results for lymphocytes, basophils, neutrophils, eosinophils, and monocytes.

## 1 Introduction

Leukocytes are an important part of the body’s immune system. They are mainly made up of neutrophils, basophils, eosinophils, monocytes, and lymphocytes. The number and ratio of different types of leukocytes correspond to different diseases, so the accuracy of the leukocyte test is very important for the diagnosis of diseases. The traditional method of leukocyte testing is mainly manual microscopy, which requires two well-trained and experienced examiners to observe the morphology of leukocytes under the microscope to finalize the test results, which is not only time-consuming and inefficient but also highly subjective.^1^ With the improvement of computer performance and the increase of data volume in recent years, deep learning has also been widely applied to medical segmentation, and the proposed work is mainly based on various convolutional neural network schemes,^2^ such as SegNet,^3^^,^^4^ U-Net,^4^^,^^5^ and VGG-Unet.^6^ For the work on leukocyte segmentation, many researchers have achieved good results. Reena et al.^7^ used DeepLabv3+ architecture and ResNet-50 as a feature extraction network to segment each of the five types of leukocytes, and the final average segmentation accuracy was 96.1% and intersection-union accuracy was 92.1%; Lu et al.^8^ proposed a WBC-Net model based on UNet++ and ResNet to segment individual leukocytes, mainly by designing a context-aware feature encoder with residual blocks to extract multiscale features and introducing hybrid jump paths on dense convolutional blocks to acquire and fuse image features at different scales, which finally achieved good results on four publicly available datasets. Li et al.^9^ proposed a segmentation algorithm based on the U-Net model with dual-path and void space pyramidal pooling to achieve pixel-level segmentation of blood leukocytes, mainly by introducing a dual-path network in the feature encoder to extract multiscale features of leukocytes in images and using a void space pyramidal pooling module to enhance the feature extraction capability of the network, and the research results show that its mIoU value can reach above 0.97.

Through the current study, it can be found that the leukocyte data samples in the public dataset are small, many studies require data expansion, and only the segmentation of single leukocytes in an image is achieved. To address the above-mentioned problems, this paper proposes an improved U-net based leukocyte segmentation method. On the original U-net^5^ network, to be more applicable to the segmentation of leukocytes, this paper makes corresponding improvements in data augmentation, skip connection, and loss function. The data enhancement part uses adaptive histogram equalization-retinex (AHE-Retinex) to enhance the features of leukocytes to adapt to the blood cell images collected in various complex environments and to improve the generalization ability of the network. Convolutional block attention module (CBAM)^10^ is added in the skip connection part, according to the findings of the literature,^10^ where it is found that the best result is obtained by connecting channel attention module (CAM) and the spatial attention module (SAM) in series. This paper also adopts this scheme in the skip connection part. It enables the network to quickly locate the high-value information of features in different channels and spaces, avoiding a large number of repeated calculations of low-value information, preventing overfitting, and improving the training efficiency and generalization ability of the network. The loss function is partially replaced with a loss function combining focal loss^11^ and Dice loss,^12^ which can solve the problem of class imbalance in blood cell images.

## 2 Method

**Fig. 1:**
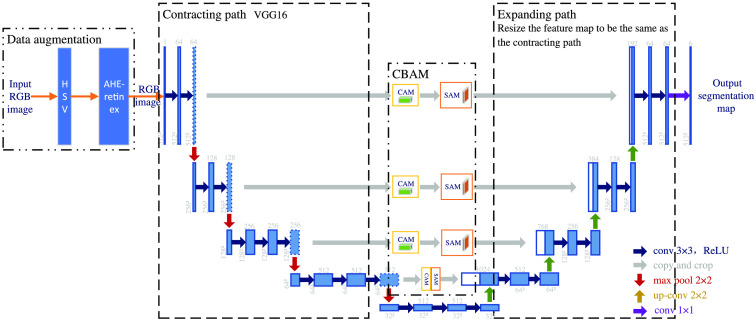
Schematic diagram of the leukocyte segmentation method.

The method in this paper mainly improves on the original U-net network with three parts, which are data enhancement, skip connection, and loss function. In this paper, U-Net is chosen as the basic model for leukocyte segmentation because U-net can get a good training effect using fewer data sets, the problem of small number of leukocyte datasets has been solved. The data enhancement preprocessing part first converts the RGB color space of the cell images into HSV (hue, saturation, value) color space and then introduces the AHE-Retinex method based on the combination of HE^13^ and retinex theory^14^ of the OpenCV platform to improve the generalization ability of the network. The processed images are passed into a contracting path with VGG16 as the backbone for feature extraction. The hopping connection part adds four CBAMs that connect CAM and SAM in series, respectively, so that the contracting path knows the channels and spatial locations to focus on when upsampling and provides high-value detail information for subsequent segmentation. The loss function is partially replaced by a loss function combining focal loss and Dice loss. The Dice loss training process focuses more on the mining of the foreground region, so it has good performance for scenes with a class imbalance in blood cell images, but the training loss is easy to be unstable in the case of small targets like leukocytes, and there are some hard-to-score samples, so the combination of focal loss can solve this problem. Figure 1 shows the overall schematic of the method in this paper.

### 2.1 Image Enhancement

**Fig. 2:**
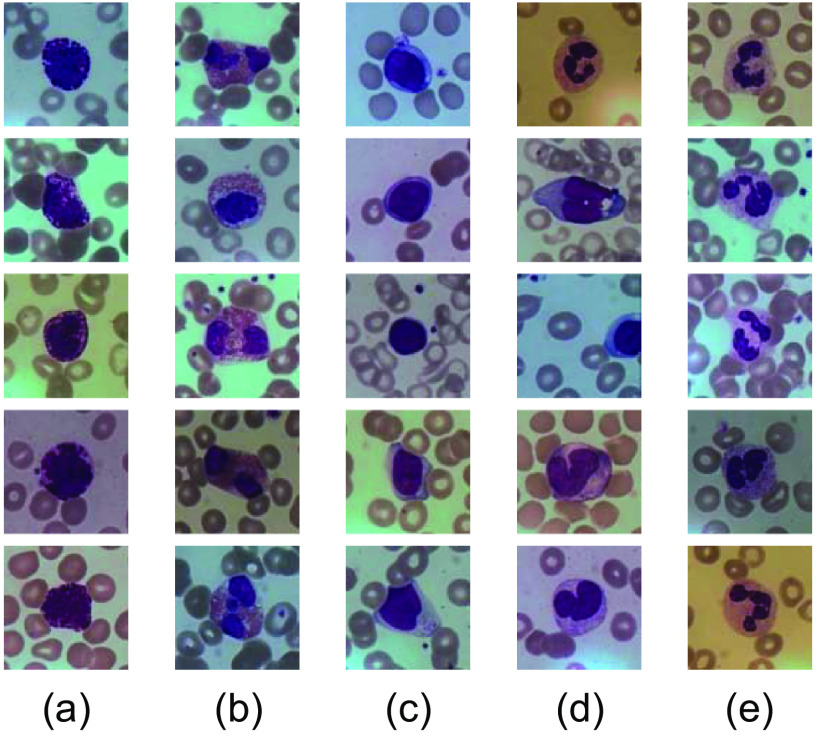
Selected leukocyte images from the BCISC dataset: (a) basophils, (b) eosinophils, (c) lymphocytes, (d) monocytes, and (e) neutrophils.

**Fig. 3:**
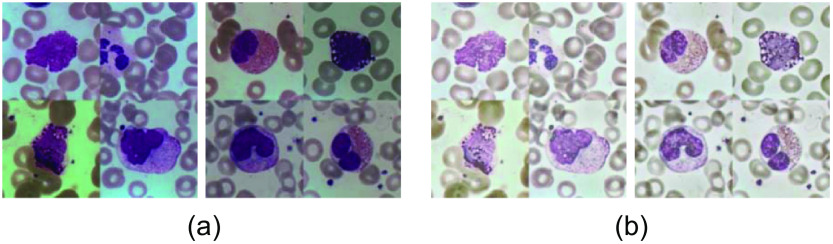
The final processed dataset: (a) original random four images and (b) final result images.

In this paper, we use the BCISC^15^ public dataset, and Fig. 2 shows some of the leukocyte images in the BCISC dataset. We find that the background color and brightness of the blood cell images are not uniform, resulting in color and brightness variability among the leukocytes as well, presumably due to the influence of lighting and the surrounding environment during the acquisition process. To eliminate this effect, we propose an AHE-Retinex correction for image pre-processing, combining AHE and multiscale Retinex with color restoration (MSRCR),^16^ which can better balance the color of leukocytes in different images and further clarify their features. In addition, one image in the BCISC dataset contains only one leukocyte, which does not meet the requirements of this paper. Therefore, in this paper, after AHE-Retinex correction of all images, 4 random images of these leukocytes were formed into 512×512  pixel size images, as shown in Fig. 3, with a total of 50 images. This will initially form the data set required for the study in this paper.

The basic idea of HE is to widen the gray levels with a large number of pixels in the image and reduce the gray levels with a small number of pixels, to achieve a clear image.^17^ The general HE input is only grayscale images, but the blood cell images in this paper are RGB color images, so this paper first performs the RGB to HSV operation on the blood cell images and HE on the V channel. Considering the fineness of the leukocyte features, the direct use of the traditional HE will lead to the loss of most of the feature information. Therefore, to solve this problem, AHE is used in this paper.

The algorithm underlying retinex theory is single scale retinex (SSR), which is implemented in the steps of Algorithm 1.

**Algorithm 1:** Single scale retinex.

Step 1: decompose the image into three channels, R, G, and B, and perform logarithmic transformation according to Eqs. (2) and (3), respectively.
Step 2: construct the Gaussian surround function and convolve the grayscale images of each channel with the Gaussian surround function respectively to obtain the illumination estimation components of the three channels.
Step 3: in the logarithmic domain, do the difference operation with the original image and the Gaussian blurred image to obtain the reflection component.
Step 4: linearly stretch or exponentially transform the result of the obtained reflection components into the image output data type.
Step 5: combine the obtained reflection component images of the three channels into one image to get the SSR-enhanced image.

**Fig. 4:**
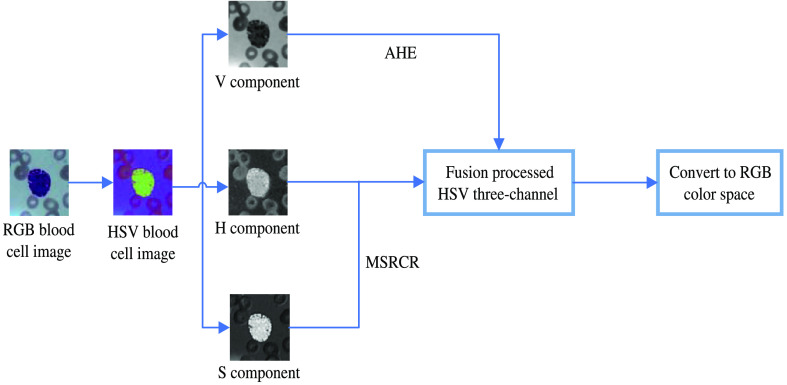
AHE-Retinex correction process.

Multiscale Retinex (MSR) is to select three scale parameters in step 2 of SSR to form three Gaussian surround functions and then convolve them separately and weigh the average to get the illumination estimation components of each channel to effectively maintain the detail and color information. MSRCR, on the other hand, is based on MSR and uses the color recovery function in step 4 to multiply with the MSR enhancement function of each channel. MSRCR is based on MSR, and the color recovery function is multiplied by the MSR enhancement function of each channel in step 4 to obtain the image enhancement reflection component of the three channels to reduce the color bias. The AHE-Retinex algorithm proposed in this paper is based on MSRCR, converting the image in RGB color space to HSV color space, and performing AHE processing on the V component. The overall algorithm processing process is shown in Fig. 4.

The MSRCR model is as follows:

$$F_{\mathrm{MSRCR}}=(x,y)=c_{i}(x,y)F_{\mathrm{MSR}}(x,y),$$ (1)

where $c_{i}(x,y)$ denotes the color recovery function and the equation satisfies

$$c_{i}(x,y)=\alpha \cdot \log[\beta \cdot \frac{S_{i}(x,y)}{\sum_{i}S_{i}(x,y)}],$$ (2)

where i∈{H,S,V}, α and β and are the offsets of the gain factor affecting the image color recovery, respectively, and are constants, and generally α takes the value of 46 and β takes the value of 125.

### 2.2 Skip-Connection

**Fig. 5:**
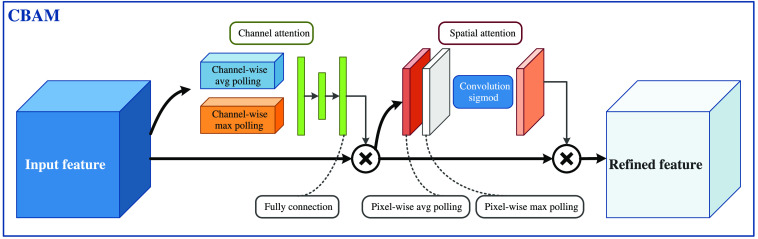
CBAM.

The key to the U-Net^5^ network structure is the skip-connection between the contracting path and the expanding path, which combines the deep high-level features from the expanding path with the shallow low-level features from the contracting path. The contracting path progressively down-samples the feature map through the pooling layer, while the expanding path up-samples the low-resolution feature map into a pixel-level segmentation result map. To compensate for the information lost in downsampling during the encoding stage, the U-net network uses a hopping connection to fuse the feature maps at the corresponding positions in the two processes, so that the expanding path can obtain more high-resolution information when up-sampling, and thus better recover the information in the original image. Therefore, adding the attention mechanism to the skip connection enables the network to pay more attention to the important parts of the shallow features in the upsampling process at each level, and through the fusion of the shallow features with the deep features, the network can retain more high-resolution detail information contained in the shallow feature maps, thus improving the image segmentation accuracy. Then considering that the spatial information and the edge information in the image are very important in the leukocyte segmentation task, this paper adopts a CBAM^10^ approach that connects CAM and SAM in tandem, and its implementation schematic is shown in Fig. 5.

It can be seen that CBAM contains two separate submodules, the CAM and the SAM, which perform channel and spatial attention, respectively. This not only reduces parameters and the amount of calculations, but also ensures that it can be integrated into existing network architectures as a plug-and-play module.

**Fig. 6:**
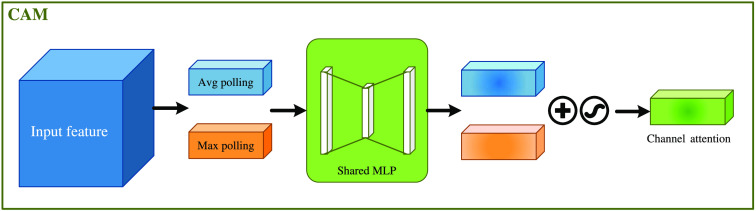
CAM.

The CAM is shown in Fig. 6. The input feature map (H×W×C) are subjected to global max pooling and global average pooling based on width and height, respectively, to obtain two 1×1×C feature maps. Then, they are fed into a two-layer neural network (MLP). The number of neurons in the first layer is C/r (r is the reduction rate), the activation function is Relu, and the number of neurons in the second layer is C. This two-layer neural network is shared. Then, the MLP output features are subjected to element-wise summation operation and then sigmoid activation operation to generate the final channel attention feature. Finally, the obtained channel attention feature and the input feature map are multiplied element-wise to generate the input features required by the SAM.

That is, CAM compresses the feature map in the spatial dimension to obtain a one-dimensional vector and then operates on it. When compressing in the spatial dimension, not only the average pooling but also the max pooling is taken into account. The average pooling and max pooling can be used to aggregate the spatial information of the feature map, send it to a shared network, compress the spatial dimensions of the input feature map, and sum up element by element to produce a channel attention map. The average pooling has feedback for every pixel point on the feature map, while the max pooling has feedback for gradients only where the response is greatest in the feature map when performing gradient backpropagation calculations.

**Fig. 7:**
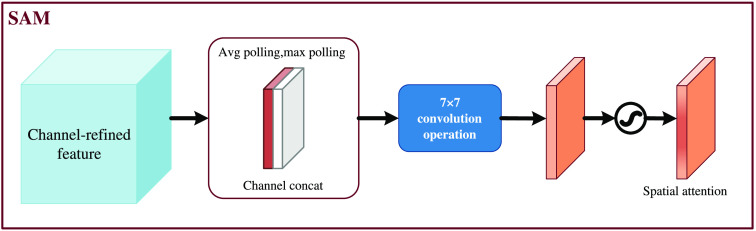
SAM.

The SAM is shown in Fig. 7. The feature map output from the CAM is used as the input feature map for this module. First, global max pooling and global average pooling based on channels are performed to obtain two H×W×1 feature maps. Then, these two feature maps are concatted based on channels (channel stacking). Then, the spatial attention feature is generated by sigmoid. Finally, this feature is multiplied by the input feature to obtain the final generated feature.

Again, the SAM is to compress the channels, and the average pooling and the max pooling are performed in the channel dimension. Max pooling operates by extracting the maximum value on the channel, and the number of extractions is height × width; the average pooling operates by extracting the mean value on the channel, and the number of extractions is also height × width.

### 2.3 Loss Function

The use of loss functions usually takes into account the characteristics of the dataset. Usually there is a problem of extreme class imbalance in the blood cell images, which means that the number of different types of leukocytes can vary greatly, for example, the number of eosinophils and basophils is very small. The small number of samples produces fewer loss values, forming a hard-to-score sample; while the majority of the total loss function input parameters are easy-to-score samples, thus making the optimization direction of the model (the gradient descent direction of the loss function) not as desired in this paper. For this problem, the focal loss function can be used, with the following equation:

$$L_{fl}=-{\left(1-p_{t}\right)}^{\gamma }\;\log(p_{t}),$$ (3)

$L_{fl}$ is an improvement based on the cross-entropy (CE) loss function, which reduces the weight of a large number of simple negative samples in the training, so focal loss is equivalent to increasing the weight of hard-to-score samples in the loss function, which makes the loss function favor hard-to-score samples and helps to improve the accuracy of hard-to-score samples. Among them, $p_{t}$ reflects the proximity to category y. The larger $p_{t}$ indicates the closer to category y, i.e., the more accurate the classification. $p_{t}$ also reflects the ease of classification. The larger $p_{t}$ indicates the higher confidence of classification, representing the easier to classify the samples; the smaller $p_{t}$, the lower confidence of classification, representing the harder to classify the samples. γ>0 is the adjustable factor, which reduces the loss of easily classified samples, making more attention to difficult and misclassified samples. Experimentally, γ=2 is optimal.

In addition, in the experiments of leukocyte segmentation, we found that the nuclei of different types of leukocytes can be easily segmented because of their different morphologies. However, the cytoplasm was more difficult to be segmented. First, the cytoplasm of leukocytes is similar in color to red blood cells in the stained blood cell images, so it is easy to segment the red blood cells together. Second, the cytoplasm of different types of leukocytes is also similar, which may cause confusion. Therefore, in this paper, considering the relevant a priori knowledge that the cytoplasm of neutrophils, eosinophils, and basophils contains special staining granules after staining with Richter’s dye, while monocytes and lymphocytes have no cytoplasmic granules, Dice loss is used to further regulate the loss function by the different granules on the cytoplasm of leukocytes. The equation of Dice loss is as follows:

$$L_{dl}=1-\text{Dice},$$ (4)

$$\text{Dice}=\frac{2TP}{\mathrm{FP}+2\mathrm{TP}+\mathrm{FN}},$$ (5)

$L_{dl}$ is designed to cope with the scenario of imbalance between positive and negative samples in semantic segmentation. Dice loss is able to focus on very small areas of the target. The training can focus more on the particles on different kinds of leukocytes. Dice is an ensemble similarity measure function, which is usually used to calculate the similarity of two samples, taking values in the range of [0,1], the larger the value means the more similar.

In general, the direct use of Dice loss will adversely affect the back propagation and easily make the training unstable, while this paper then combines focal loss to solve the class imbalance problem of white blood cells in blood cell images. The loss function in this paper is as follows:

$$L=L_{dl}+L_{fl},$$ (6)

## 3 Experiment

To verify the accuracy and effectiveness of the improved U-net in leukocyte five classification segmentation, this paper compares it with three classical segmentation methods: U-Net,^5^ DeepLabv3+,^18^ and PSPnet.^19^ To further verify its robustness, experiments are conducted on four different publicly available datasets. And the proposed method in this paper is compared with four methods, FCN,^20^ U-net,^5^ WBC-Net,^8^ and LeukocyteMask.^15^

### 3.1 Datasets

**Fig. 8:**
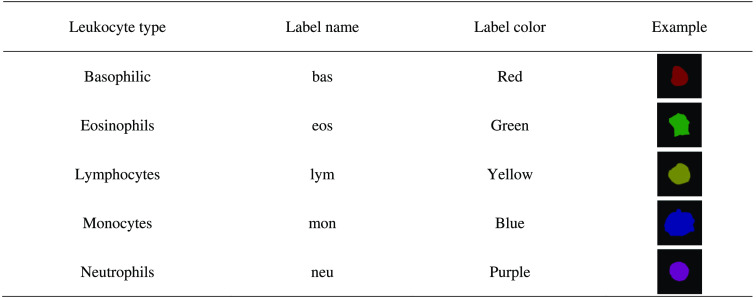
Labeling colors of leukocytes.

In this paper, we used four datasets named Dataset1, Dataset2, Dataset3, and Dataset4. Dataset1^15^ is from the Third People’s Hospital of Fujian Province, China, and contains 268 individual leukocyte images of 256×256  pixels in size (51 neutrophils, 54 eosinophils, 56 basophils, 54 monocytes, and 53 lymphocytes), which were processed into 50 multiple leukocyte images of 512×512  pixels in size to suit the experimental purpose of this paper. Dataset2^21^ was collected by Jiangxi Dekang Technology Co., Ltd., in China and contained 300 individual leukocyte images of 120×120  pixels (176 neutrophils, 22 eosinophils, 1 basophils, 48 monocytes, and 53 lymphocytes). Dataset3^21^ was published in the CellaVision blog and contains 100 individual leukocyte images of 300×300  pixels in size (30 neutrophils, 12 eosinophils, 3 basophils, 18 monocytes, and 37 lymphocytes). Dataset4,^22^ called LISC, is a peripheral blood image of healthy subjects and contains 242 individual leukocyte images of 720×576  pixels in size (50 neutrophils, 39 eosinophils, 53 basophils, 48 monocytes, and 52 lymphocytes). The images in Dataset2 were captured under fast staining conditions, while the images in the other three datasets were captured under standard staining conditions. In this paper, these datasets were re-labeled with different colors to indicate different types of leukocytes, as shown in Fig. 8.

### 3.2 Evaluation Metrics

To evaluate the segmentation performance and robustness of the image, this paper introduces the concept of a confusion matrix.^23^ Among them, TP is true positive, which indicates that positive samples are correctly determined as positive samples; FP is false positive, which indicates that negative samples are misjudged as positive samples; FN is false negative, which indicates that positive samples are misjudged as negative samples; and TN is true negative, which indicates that negative sample is correctly determined as a negative sample. In this paper, precision (P), recall (R), F-measure (F1), intersection over union (IoU), and accuracy (Acc) are selected. Precision and recall are mutually constrained, and it is difficult to judge the performance of segmentation from these two metrics alone, while the comprehensive metric F1 considers precision and recall, which can evaluate the performance of the algorithm more comprehensively. Among them, if the IoU obtained from each class of prediction is summed and averaged, mean intersection over union (mIoU) will be obtained. Then the higher the values of F1, IoU, Acc, and mIoU, the higher the segmentation accuracy. These indicators are defined as follows:

$$P=\frac{\mathrm{TP}}{\mathrm{TP}+\mathrm{FP}}=\frac{TN}{\mathrm{TN}+\mathrm{FN}},$$ (7)

$$R=\frac{\mathrm{TP}}{\mathrm{TP}+\mathrm{FN}}=\frac{\mathrm{TN}}{\mathrm{TN}+\mathrm{FP}},$$ (8)

$$F1=\frac{2\mathrm{PR}}{P+R},$$ (9)

$$\mathrm{IoU}=\frac{\mathrm{TP}}{\mathrm{FP}+\mathrm{TP}+\mathrm{FN}},$$ (10)

$$\mathrm{Acc}=\frac{\mathrm{TP}+\mathrm{TN}}{\mathrm{TP}+\mathrm{TN}+\mathrm{FP}+\mathrm{FN}}.$$ (11)

### 3.3 Experimental Environment

The experiments in this paper are based on the deep learning framework TensorFlow, and the experimental environment is Python 3.6. The processor is i7-9700f, the memory is 8G, the graphics card is GTX1660 Ti, and the operating system is Windows 10. The dataset used in this paper is the BCISC public dataset, provided by the Third People’s Hospital of Fujian Province, China. There is only one leukocyte on a single image in this dataset. To achieve the purpose of multiple leukocyte segmentation, four images were randomly taken for the stitching process in this paper. There are 50 multiple leukocyte images of size 512×512  pixels available. First, the sample dataset was divided into training and test sets in the ratio of 9:1. Then, the training set is fed into the U-net network for model training. In the experiment, all the training data were normalized to 512×512×3, and the pretrained weights were the initialized weights of the VGG16 network, and then the training started from generation 0. Because the training weights were used to train the VGG16 weights, to put more resources on training the network parameters of the later part, the VGG16 backbone part was first frozen and trained for 50 generations, and then all of them were thawed and continued to be trained together for 50 generations, which makes the time and resource utilization can be greatly improved. Two data samples are crawled at a time. The initial learning rate is set to $1\times 10^{-4}$.

## 4 Results

### 4.1 Accuracy Analysis

To verify the accuracy and effectiveness of the proposed method in this paper, comparison experiments were conducted on Dataset1.^15^ Three classical segmentation methods, U-Net,^5^ DeepLabv3+,^18^ and PSPnet,^19^ were used for the comparison. The comparison of evaluation metrics is shown in Table 1. From Table 1, it can be seen that the method in this paper performs better compared to the other three segmentation methods. Among the three classical segmentation methods, we found that for each category of IoU, eosinophils, lymphocytes, and monocytes differed more compared to the other two categories of cells. This is due to the fact that each of these three types of leukocytes has certain differences of its own. For example, eosinophils it has a nucleus similar to neutrophils and is easily fragmented, and their eosinophilic granules can be dispersed around the cells, which makes network segmentation somewhat difficult. In human blood, lymphocytes are mainly composed of small lymphocytes and a certain number of medium lymphocytes, then these two types of lymphocytes are morphologically different, and the network will have some difficulty in identification. Then monocytes are due to their nucleus being kidney-shaped or horseshoe-shaped and other polymorphic, the cells also have the problem of different shapes, for the network to learn there is a certain degree of difficulty. Then in this paper, data augmentation, skip connection and loss function improvement are performed for these problems. The background of the blood cell image is formed a strong contrast with the leukocyte itself by data enhancement, so that the broken eosinophils dispersed out of the eosinophils can also be well captured by the network, and the characteristics of each leukocyte can also be enhanced. Adding an attention mechanism to the skip connection can better enable the network to focus on the different features of each type of leukocyte, thus improving the discrimination ability of the network to some extent. Then changing the loss function can improve the feature weights of the hard-to-score samples, thus solving the problems caused by the hard-to-score samples. Then from the final mIoU obtained by the method of this paper, it seems that this paper basically solves the problem of appeal, and the maximum difference of mIoU for each kind of leukocytes is at 3.13%. From the value of F1, it seems that the model output of the method in this paper is the best result. The best results were obtained for neutrophils, mainly because neutrophils have the most remarkable characteristics, with rod-shaped nuclei or 2∼5 foliated nuclei and thin filaments connected between lobes, which are different from the characteristics of all other types of leukocytes.

**Table 1:** Comparison of metrics of different segmentation methods on Dataset1.

Type	Method	P (%)	R (%)	IoU (%)	F1 (%)
Basophils	U-Net^5^	83.59	84.04	75.64	83.81
	DeepLabv3+^18^	89.20	72.91	70.50	80.24
	PSPnet^19^	94.56	84.40	83.49	89.19
	Method of this paper	93.04	88.57	91.31	90.75
Eosinophils	U-Net^5^	71.27	75.80	61.56	73.47
	DeepLabv3+^18^	66.92	61.62	50.74	64.16
	PSPnet^19^	63.23	81.47	58.28	71.20
	Method of this paper	91.17	91.15	90.08	91.16
Lymphocytes	U-Net^5^	89.03	63.89	62.72	74.39
	DeepLabv3+^18^	64.99	57.46	47.38	60.99
	PSPnet^19^	93.69	64.94	65.22	76.71
	Method of this paper	92.71	80.26	88.94	86.04
Monocytes	U-Net^5^	75.65	75.11	63.99	75.38
	DeepLabv3+^18^	72.06	59.32	51.73	65.07
	PSPnet^19^	83.64	67.91	62.95	74.96
	Method of this paper	86.32	92.60	89.24	89.35
Neutrophils	U-Net^5^	81.22	82.33	72.66	81.77
	DeepLabv3+^18^	64.51	96.64	66.60	77.37
	PSPnet^19^	72.25	96.00	73.14	82.45
	Method of this paper	92.46	95.88	92.07	94.14

**Fig. 9:**
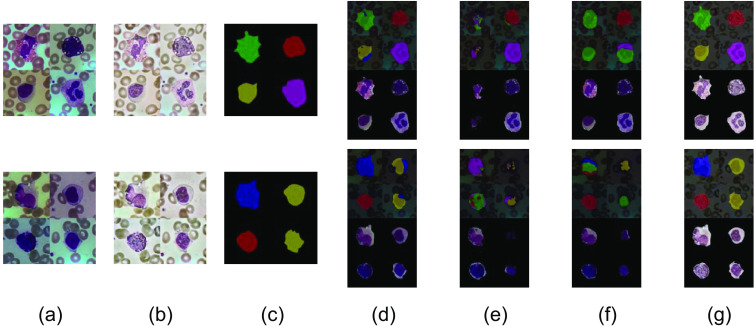
Comparison of segmentation results of different segmentation methods on Dataset1 dataset: (a) original image, (b) AHE-Retinex processed image, (c) masked image, (d) U-Net network segmentation results, (e) DeepLabv3+ network segmentation results, (f) PSPnet network segmentation results, and (g) segmentation results of this method.

Figure 9 shows the comparison of the style results of three classical segmentation methods, U-Net,^5^ DeepLabv3+,^18^ and PSPnet,^19^ and the method in this paper. Among them, Fig. 9(a) shows the original image stitched from four random images in the original Dataset1; Fig. 9(b) shows the image after AHE-Retinex processing; Fig. 9(c) shows the masked image, which serves as a comparison with the final segmentation result; and Fig. 9(d) shows the segmentation result of U-Net network. According to the results, U-Net network has better segmentation effect for leukocytes, but there is evidence of misclassification problems. Since the cytoplasm of lymphocytes and monocytes does not have granules and other features, the U-net network is confusing for the cytoplasm of lymphocytes and monocytes. Figure 9(e) shows the segmentation result of DeepLabv3+ network, which is poor for leukocyte segmentation and has the problems of missing and mis-detecting; most of the leukocytes cannot be identified and segmented. The two leukocytes on the right can be segmented in the first image because of the obvious and unique characteristics of basophils (containing basophilic granules) and neutrophils (with rod-shaped nuclei or 2 to 5 lobes and thin filaments between lobes). Figure 9(f) is the segmentation result of PSPnet network; the edges of the leukocytes segmented by this network are smooth, and the original shapes of most leukocytes are lost. Also, there are serious problems of leakage and false detection. Figure 9(g) is the segmentation result of the method in this paper. The result graph can show more intuitively that the segmentation effect of the method proposed in this paper is better, and not only can it accurately segment different kinds of leukocytes but also can segment the edge shape of leukocytes.

### 4.2 Robustness Analysis

To verify the robustness of the proposed method in this paper, four datasets are used in this paper to conduct comparison experiments on different methods. The datasets we used are Dataset1^15^, Dataset2,^21^ Dataset3,^21^ and Dataset4,^22^ which are all four datasets with only one leukocyte present on one image; the compared methods are FCN,^20^ U-Net,^5^ WBC-Net,^8^ and LeukocyteMask^15^ and the proposed method in this paper, respectively. The evaluation metrics are P, R, F1, mIoU, and Acc, and the data comparison is shown in Table 2. From Table 2, it can be seen that both in F1 and mIoU and in Acc, the approach in this paper achieves good performance compared to the other four approaches. Although it performs poorly on Dataset4 compared to the other three datasets, the other four approaches do not achieve better accuracy either. We studied these four datasets and found that the range of leukocytes on the blood cell images in Dataset4 was small and the leukocyte features were blurred. Considering the four datasets together, the model output of this paper’s method is better and the segmentation accuracy achieves good results. Overall, the performance of this paper’s method for leukocyte segmentation is the best.

**Table 2:** Comparison of the metrics of different segmentation methods on different datasets.

Dataset	Method	P (%)	R (%)	F1 (%)	mIoU (%)	Acc (%)
Dataset1	FCN^20^	97.05	96.84	96.94	96.48	99.51
	U-Net^5^	97.08	95.44	96.25	95.74	99.63
	WBC-Net^8^	98.47	97.26	97.86	97.41	99.87
	LeukocyteMask^15^	97.24	97.01	97.12	97.02	99.91
	Method in this paper	99.13	98.60	98.86	98.77	99.96
Dataset2	FCN^20^	96.92	96.59	96.75	96.37	98.88
	U-Net^5^	97.40	96.91	97.15	96.99	98.35
	WBC-Net^8^	98.89	98.78	98.83	98.29	99.27
	LeukocyteMask^15^	98.06	97.91	97.98	97.14	99.05
	Method in this paper	99.15	98.79	98.97	99.73	99.99
Dataset3	FCN^20^	95.95	95.11	95.53	95.45	99.33
	U-Net^5^	96.89	95.62	96.25	96.17	99.36
	WBC-Net^8^	97.98	97.85	97.91	97.69	99.68
	LeukocyteMask^15^	97.43	97.91	97.17	97.04	99.92
	Method in this paper	98.99	98.87	98.93	98.34	99.93
Dataset4	FCN^20^	92.15	91.24	91.69	91.66	98.98
	U-Net^5^	92.27	90.85	91.55	91.35	99.03
	WBC-Net^8^	92.74	91.18	91.95	91.79	99.80
	LeukocyteMask^15^	96.64	95.49	96.06	95.58	99.22
	Method in this paper	98.64	98.01	98.32	96.53	99.83

### 4.3 Ablation Experiment

A total of three partial improvements are proposed in this paper, which is summarized in Table 3, with mIoU and Acc selected as comparative metrics. Among them, the original network is a U-net with a loss function of the CE loss function, and both metrics are improved to a great extent after adding the AHE-Retinex image enhancement. With the addition of CBAM or replacing the loss function with a combination of Dice loss and focal loss, another improvement was achieved, indicating that the features of leukocytes in blood cell images are more complex and difficult to segment. Finally, all three improvements were used in U-net for leukocyte segmentation, and a good result was obtained. mIoU could reach 91.89% and Acc could reach 99.53%.

**Table 3:** Ablation experiment.

CE loss	AHE-Retinex	CBAM	Dice loss + focal loss	mIoU (%)	Acc (%)
√				72.58	95.31
			√	85.06	96.11
√		√		83.57	95.92
√	√			80.40	95.80
√	√	√		87.89	96.48
	√		√	88.54	97.59
	√	√	√	91.89	99.53

**Fig. 10:**
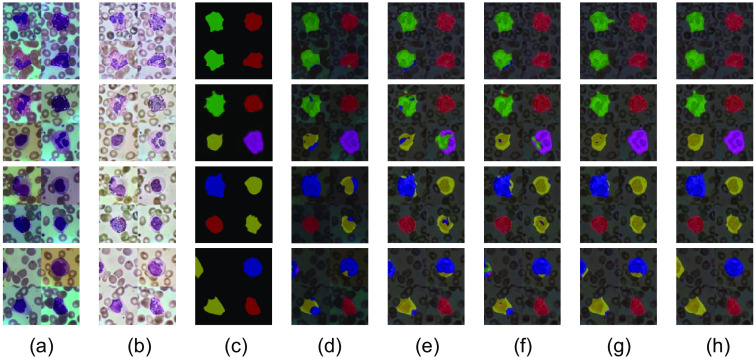
Leukocyte segmentation results from the ablation experiment. The purpose of the ablation experiment is to verify the validity of the proposed improvement points, and the fusion of the predicted result of the masked and original images gives a more effective indication of the effectiveness of the improvement points. The leukocyte image segmented by removing the background does not illustrate the problem, e.g., even if the edge parts of monocytes and lymphocytes are confused, as long as there are masked parts they can be segmented intact. So in comparison to Fig. 9, part labels (d), (e), (f), (g), and (h) in Fig. 10, there are no additional segmented leucocyte images: (a) original image, (b) AHE-Retinex processed image, (c) masked image, (d) segmentation result of U-net network with loss function of CE, (e) segmentation results of the data after AHE-Retinex processing, (f) segmentation results of AHE-Retinex with CBAM added on top of it, (g) the segmentation result of replacing the loss function with a combination of Dice and focal functions on the basis of AHE-Retinex, and (h) segmentation results of the method in this paper.

The final leukocyte segmentation results are shown in Fig. 10(a) shows the original image of any three stitched images in the dataset; Fig. 10(b) shows the three images after AHE-Retinex processing; Fig. 10(c) shows the label mask corresponding to these three images, which serves as a comparison with the final segmentation results; and Fig. 10(d) shows the segmentation results of the U-net with loss function CE. According to the results, the original network has the problem of misclassification and inaccurate judgment for the segmentation of leukocytes. Since there are no features such as granules on the cytoplasm of both lymphocytes and monocytes, the U-net with loss function CE is confusing for the cytoplasmic judgments of lymphocytes and monocytes. The lymphocytes in the fourth image were misjudged as monocytes; Fig. 10(e) shows the segmentation result of the data after AHE-Retinex processing. Since the characteristics of each type of leukocyte are clearer, some improvement was achieved in the problem of cytoplasmic confusion, but a small number of misjudgments occurred, mainly in the second image where the nuclei of neutrophils were misjudged as eosinophils, because the nuclei of eosinophils and neutrophils are too similar, and the network is more likely to confuse the two when the characteristics of leukocytes are clear. The misjudgment situation of the fourth image still exists; Fig. 10(f) is the result of the segmentation of CBAM added to AHE-Retinex. The results show that the nucleus confusion problem in Fig. 10(e) still exists but has been somewhat improved, greatly reducing the range of misclassified pixels. The percentage of monocytes and lymphocytes being misclassified is also decreasing, but the problem still exists in the third and fourth images; Fig. 10(g) is the segmentation result of replacing the loss function with a combination of Dice and focal functions based on AHE-Retinex. It is found through the results that the change of the loss function greatly improves the prediction results of the hard-to-score samples. The medium and fine granular cells in the second figure can be detected and segmented completely. Then for the hard-to-score lymphocytes and monocytes also achieved some improvement, the misclassified range in the third graph basically no longer exists, and the misclassified range in the fourth graph has been significantly reduced; Fig. 10(h) is the segmentation result of the proposed method in this paper, which combines all three methods together. The problems in Figs. 10(d) and 10(e) are solved, the cytoplasmic misclassification problem is solved, and the most difficult lymphocytes in the fourth graph are also segmented, resulting in a better leukocyte segmentation result.

### 4.4 Additional Experiment

**Fig. 11:**
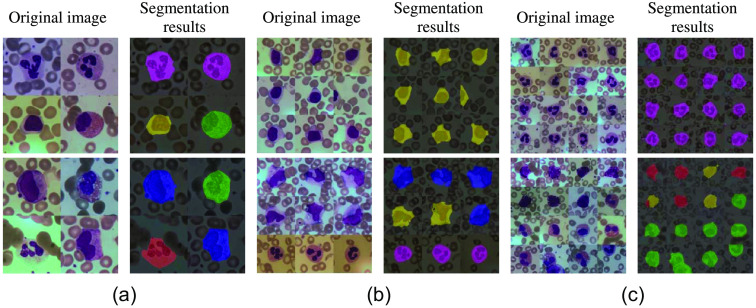
Segmentation results of different numbers of leukocytes. (a) The original image and segmentation results with 4 leukocytes, (b) original figure and segmentation results with 9 leukocytes, and (c) the original image and segmentation results with 16 leukocytes.

To verify the generality and effectiveness of the method proposed in this paper, dataset1 was randomly combined again in this paper, so that 4, 9, and 16 leukocytes were present on the 512×512 size image. The final segmentation results are shown in Fig. 11.

From the experimental results, we found that the segmentation accuracy is higher on images containing fewer leukocytes, while a small amount of cytoplasmic misclassification occurs as the number of leukocytes increases, but in general it does not segment the whole leukocyte. As shown in Fig. 11(a), when using the 2×2 size stitching, the image contains four leukocytes, and the experimental results show that its segmentation accuracy is high and it is suitable for segmentation containing multiple types of leukocytes. When the 3×3 size is used, the number of leukocytes increases to 9, as shown in Fig. 11(b), and good segmentation results can be achieved for both similar and different types of leukocytes. However, when the number of leukocytes in the image increased to 16 using the 4×4 size stitching, there was an error in the performance of multiclass leukocyte segmentation. As shown in the second image in Fig. 11(c), the cytoplasm of the second leukocyte on the leftmost side of this image shows mis-segmentation and it is not difficult to find that although there is a small part of mis-segmentation, it is not significant for the overall segmented image of leukocytes. Also, the first image in Fig. 11(c) shows that the model proposed in this paper has a high accuracy rate for multiple numbers of the same type of leukocytes. Therefore, through the above experimental analysis, the method proposed in this paper has some generality and validity.

In addition, in this paper, the network of contracting paths was changed to ResNet50^24^ to further validate the generalizability of the improved U-net, and all the improvement points mentioned in the previous section (image enhancement, CBAM, and loss function) were left unchanged. Table 4 shows a comparison of the mIoU and Acc data for the VGG16 and ResNet50 networks. resNet50 also achieved good results, with a segmentation accuracy of 95.18%. However, directly replacing the contracting path with the ResNet50 network did not achieve better results than VGG16.

**Table 4:** Network comparison experiments for contracting path.

Contracting path	mIoU (%)	Acc (%)
VGG16	91.89	99.53
ResNet50	86.34	95.18

## 5 Conclusion

In this paper, an improved U-net-based leukocyte segmentation method is proposed. To be more applicable to leukocyte segmentation, this paper focuses on three parts of the original U-net network, namely data enhancement, skip connection, and loss function. The data enhancement part is based on the OpenCV platform, which performs adaptive HE operation on the V component of the image and MSRCR operation on the H and S components to improve the generalization ability of the network and make the features of each leukocyte clearer to improve the robustness during training. Four CBAMs that connect CAM and SAM in series are added in the skip connection part, respectively, so that the contracting paths know the channels and spatial locations that need attention when upsampling and provide high-value detail information for subsequent segmentation to realize the segmentation work of multiclass leukocytes. The loss function is partially replaced by a loss function combining focal loss and Dice loss. Dice loss can solve the problem of class imbalance in the blood cell images, while there are some hard-to-score samples in leukocytes, so focal loss is combined. The segmentation of multiple leukocytes using the method of this paper can achieve 99.53% Acc.

## Code, Data, and Materials Availability

Previously reported [BCISC] data were used to support this study and are available at https://github.com/fpklipic/BCISC. These prior datasets are cited at relevant places within the text as Ref. 15. Previously reported [Cellavision] data were used to support this study and are available at https://data.mendeley.com/datasets/w7cvnmn4c5/1. These prior datasets are cited at relevant places within the text as Ref. 21. Previously reported [LISC] data were used to support this study and are available at http://users.cecs.anu.edu.au/~hrezatofighi/Data/Leukocyte%20Data.htm. These prior datasets are cited at relevant places within the text as Ref. 22.
